# Genotype by environment and genotype by yield*trait interactions in sugar beet: analyzing yield stability and determining key traits association

**DOI:** 10.1038/s41598-023-51061-9

**Published:** 2024-01-03

**Authors:** Mahdi Hassani, Seyed Bagher Mahmoudi, Ali Saremirad, Dariush Taleghani

**Affiliations:** Sugar Beet Seed Institute (SBSI), Agricultural Research, Education and Extension Organization (AREEO), Karaj, Iran

**Keywords:** Plant breeding, Plant sciences

## Abstract

The genotype by environment interaction significantly influences plant yield, making it imperative to understand its nature for the creation of breeding programs to enhance crop production. However, this is not the only obstacle in the yield improvement process. Breeders also face the significant challenge of unfavorable and negative correlations among key traits. In this study, the stability of root yield and white sugar yield, and the association between the key traits of root yield, sugar content, nitrogen, sodium, and potassium were examined in 20 sugar beet genotypes. The study was conducted using a randomized complete block design with four replications over two consecutive years across five locations. The combined analysis of variance results revealed significant main effects of year, location, and genotype on both root yield and white sugar yield. Notably, two-way and three-way interactions between these main effects on root yield and white sugar yield resulted in a significant difference. The additive main effect and multiplicative interaction analysis revealed that the first five interaction principal components significantly impacted both the root yield and white sugar yield. The linear mixed model results for root yield and white sugar yield indicated that the genotype effect and the genotype by environment interaction were significant. The weighted average absolute scores of the best linear unbiased predictions biplot demonstrated that genotypes 20, 4, 7, 2, 16, 3, 6, 1, 14, and 15 were superior in terms of root yield. For white sugar yield, genotypes 4, 16, 3, 7, 5, 1, 10, 20, 2, and 6 stood out. These genotypes were not only stable but also had a yield value higher than the total average. All key traits, which include sugar content, sodium, potassium, and *alpha amino* nitrogen, demonstrated a negative correlation with root yield. Based on the genotype by yield*trait analysis results, genotypes 20, 19, and 16 demonstrated optimal performance when considering the combination of root yield with sugar content, sodium, *alpha amino* nitrogen, and potassium. The multi-trait stability study, genotype 13 ranked first, and genotypes 10, 8, and 9 were identified as the most ideal stable genotypes across all traits. According to the multi-trait stability index, genotype 13 emerged as the top-ranking genotype. Additionally, genotypes 10, 8, and 9 were recognized as the most stable genotypes.

## Introduction

Sugar beet, due to its diverse applications and nutritional benefits, holds significant importance^1–4^. It is packed with a high concentration of sucrose^5^, making it a substantial source of sugar. This makes it the world second most cultivated crop for white sugar production, after sugarcane^6^. With the growing population^7^ and consequent increase in societal needs, plant breeders are striving to enhance the yield and quality of sugar beet^8–11^. However, the crop yield is determined by the genotype, environment, and genotype by environment interaction (GEI); Given its polygenic nature, the sugar beet yield is heavily influenced by environmental factors. As a result, significant fluctuations in its yield can lead to instability in production^9^; Therefore, maintaining the yield stability of genotypes has become a critical aspect of the breeding process^12^. The GEI plays a crucial role in determining the yield stability of genotypes across different environments^8,13,14^. This phenomenon shows that the environment in which a trait is expressed can affect how a given genotype (gene arrangement) affects that trait^15,16^. In layman's words, this phenomenon explains how the expression of genetic features can change based on certain environmental conditions^9^, resulting in various phenotypic outcomes for a genotype with the same gene organization in various environments. This complexity presents a significant challenge to breeding for yield increase. To address this, breeders can identify genotypes that consistently perform well in various environmental conditions by extensively testing the genotypes in locations with a range of environmental conditions^13^. This approach aids in the selection of genotypes with broad adaptability and stability^11,17^, ultimately leading to stability in yield increase.

Sophisticated statistical methods have been developed to study and expose patterns of GEI comprehensively. The model known as additive main effects and multiplicative interaction (AMMI) is a significant statistical method that combines the analysis of variance with principal component analysis^18^. The model plays a crucial role in examining the impact of GEI and visualizing its patterns. This model aids in identifying stable genotypes across varying environments^19^. However, the AMMI model has its limitations^20,21^. It assumes that the effects of genotypes and environments are additive, which may not always be true^22^. This assumption can lead to skewed estimates of genotype and environment effects. Furthermore, this model is designed to explore linear associations between genotype and environment, but non-linear associations may exist^23^. The best linear unbiased predictions (BLUP) model addresses some of the AMMI model limitations, particularly regarding non-additivity and the analysis of the linear mixed model (LMM) structure. The BLUP model provides estimations of the yield average of high-performing genotypes. To leverage the strengths of both the AMMI and BLUP models, an index called the stability index of the weighted average absolute scores of BLUPs (WAASB) was introduced^20^. This index is used to select superior genotypes. The combination of the AMMI model's power and the BLUP model's prediction accuracy enhances the efficiency of both models in studying GEI^20^. However, when introducing a new cultivar, both the yield stability and the yield value are considered; As such, the WAASBY index was introduced based on the WAASB and yield value. This index as a measure of phenotypic stability and product yield allows for the selection of genotypes with high yield potential while reducing GEI. These selected genotypes can then be included in the cultivar introduction program.

The GEI is not the only hurdle in the breeding process aimed at enhancing yield^24^. Another significant obstacle breeders encounter is the unfavorable and negative correlation among crucial traits^25–27^. In sugar beet, the white sugar yield (WSY) is determined by a combination of quantitative and qualitative traits such as root yield (RY), sugar content (SC), *alpha amino* nitrogen (N), sodium (Na^+^) and potassium (K^+^)^28–30^. The correlation among these traits ranges from negative to positive^31,32^, adding complexity to sugar beet breeding programs^10^. Negative correlation among traits can cause a dilemma; when one trait is selected for improvement, it may unintentionally lead to a decrease in another trait^33^. Therefore, breeders must find a way to improve multiple traits while managing the negative correlations among them. Breeders can mitigate these negative effects by employing techniques like tandem selection, independent culling, and index selection approaches, thereby improving multiple traits at once^24,34,35^. However, these techniques have their own challenges and can potentially lead to biased results^24,36^. The responsibility of assigning weights to each trait in the index selection and determining the cutoff points for each trait in independent culling lies with the breeder. Consequently, the assigned weights and cutoff points can vary from one researcher to another and over time^24^. The Genotype by yield*trait (GYT) biplot graphic method has been proposed as a solution to these problems. This method assumes that yield is the most essential trait, and other key traits are only significant when they correlate with high yield and the superiority of a genotype should be evaluated based on its ability to combine high yield with other traits^24^. In essence, yield is the sole trait that can determine a genotype efficiency, and other traits are only valuable when they correlate with suitable yield levels. Thus, when selecting superior cultivars, the combination of yield with traits is more important than assessing cultivars based on each trait. An ideal genotype is one that achieves the optimal level of each trait, balancing the negative effect of trait correlations and demonstrating suitable yield across different environments^36^.

In a recent study by Olivoto et al.^37^, they introduced a new index called the multi-trait stability index (MTSI). This index is highly efficient in selecting stable genotypes in multi-environment experiments based on multiple traits^8,11,38,39^. It considers the correlation structure among traits and provides a unique selection process that is easy to interpret^37^. As a result, breeders who aim to simultaneously select for multiple traits can find it useful. The main objective of the study was to identify superior genotypes from a pool of promising sugar beet genotypes. In this regard, the study examined the influence of GEI on the yield stability of sugar beet genotypes. It also analyzed the correlation among critical traits that affect yield and finally, the superior sugar beet genotypes were selected.

## Materials and methods

### Plant materials

For this study, a total of 20 sugar beet genotypes were utilized, comprising 16 recently developed hybrids and four control cultivars (Table 1). The hybrids were developed at the Sugar Beet Seed Institute (SBSI), Karaj, Alborz, Iran, with the specific aim of introducing the resistance gene for rhizomania disease into genotypes that exhibited favorable quantitative and qualitative traits. From this set of hybrids, the 16 superior hybrids, as determined by their quantitative and qualitative traits, were chosen for further evaluations. Prior to the evaluations, the hybrids resistance against rhizomania disease was verified through both field and molecular assessments.

**Table 1:** List of examined sugar beet genotypes and control cultivars.

Genotype code	Genotype	Genotype code	Genotype
1	SBSI 124	11	SBSI 134
2	SBSI 125	12	SBSI 135
3	SBSI 126	13	SBSI 136
4	SBSI 127	14	SBSI 137
5	SBSI 128	15	SBSI 138
6	SBSI 129	16	SBSI 068
7	SBSI 130	17	Shokoufa (check)
8	SBSI 131	18	Arta (check)
9	SBSI 132	19	Azare (check)
10	SBSI 133	20	Rosire (check)

### Research sites and experimental design

Phenotypic assessments of experimental genotypes were conducted over two consecutive crop years (2020 and 2021) at five agricultural research stations located in Hamadan, Karaj, Mashhad, Miandoab, and Shiraz. These selected sites differed in terms of altitude, latitude and longitude (Table 2), atmospheric temperature and precipitation (Tables 3), and Physical and chemical characteristics of soil (Table 4). The experiments at each research station were carried out using a randomized complete block design with four replications. Each genotype was planted in a separate plot measuring 30 m^2^, consisting of six cultivation rows with a length of 10 m and a distance of 50 cm interrow. The experiments were conducted from April 10th to April 20th in both years. Throughout the growing season, weed control, irrigation, fertilizer application, and other field management activities were performed based on the recommendations of experts. Additionally, regular monitoring and prevention of pests and diseases specific to sugar beet were conducted at each research station. From October 20th to October 30th in both 2 years, the roots of four rows in the middle of the cultivation area were harvested, counted, and weighed, with one meter excluded from the beginning and end of the rows.

**Table 2:** Characteristics of environmental conditions at experimental research stations.

Environment code	Year	Location of the research station	Altitude (m)	Latitude	Longitude
E1	2020	Karaj, Alborz, Iran	1312	35°55′N	50°54′E
E2	2021				
E3	2020	Mashhad, Khorasan Razavi, Iran	1316	36°30′N	59°37′E
E4	2021				
E5	2020	Miandoab, West Azarbaijan, Iran	1296	36°58′N	46°05′E
E6	2021				
E7	2020	Shiraz, Fars, Iran	1484	29°32′N	52°36′E
E8	2021				
E9	2020	Hamedan, Iran	1818	34°47′N	48°30′E
E10	2021

**Table 3:** Weather characteristics at the experimental research stations.

Location of the research station	Parameter	April	May	June	July	August	September	October
Karaj, Alborz, Iran	Temperature (°C)	9.40–19.40	15.00–25.55	19.42–31.74	21.70–33.92	21.10–33.34	16.73–28.90	10.50–21.11
	Rainfall (mm)	20.30	10.11	2.50	2.52	2.51	15.22	30.53
Mashhad, Khorasan Razavi, Iran	Temperature (°C)	9.40–21.61	14.44–27.23	18.94–32.85	21.13–34.47	18.90–33.33	13.90–28.96	8.32–22.22
	Rainfall (mm)	15.21	7.60	2.53	0.00	0.00	2.51	2.52
Miandoab, West Azarbaijan, Iran	Temperature (°C)	6.75–18.90	11.18–25.00	16.12–31.31	20.00–34.41	19.48–34.46	14.40–29.40	8.93–21.70
	Rainfall (mm)	27.91	20.32	7.61	2.50	2.50	5.00	17.81
Shiraz, Fars, Iran	Temperature (°C)	10.00–23.92	15.60–30.53	30.53–35.50	21.74–37.21	20.50–36.10	16.10–32.81	10.50–26.73
	Rainfall (mm)	15.22	5.00	2.52	2.52	2.51	0.00	5.00
Hamedan, Iran	Temperature (°C)	5.55–17.20	10.50–23.33	15.00–30.01	18.92–33.83	17.80–32.81	12.81–28.34	7.80–21.11
	Rainfall (mm)	27.93	12.71	5.00	2.54	5.00	10.62	22.81

**Table 4:** Physical and chemical characteristics of soil at the experimental research stations.

Location of the research station	Year	pH	EC (ds.m^-1^)	O.C. (%)	P (ppm)	K (ppm)	Clay (%)	Silt (%)	Sand (%)	Texture
Karaj, Alborz, Iran	2020	7.21	0.53	0.74	8.24	596.00	37.30	41.40	21.30	Clay-loam
	2021	7.30	0.77	1.01	1.97	398.00	37.30	41.40	21.30	Clay-loam
Mashhad, Khorasan Razavi, Iran	2020	7.90	1.55	0.70	12.00	261.00	21.00	57.00	20.00	Silty-clay-loam
	2021	7.65	1.67	0.75	12.10	296.00	16.00	36.00	48.00	Silty-loam
Miandoab, West Azarbaijan, Iran	2020	7.30	0.83	49.00	13.00	402.00	65.00	58.00	25.00	Silty-loam
	2021	7.10	0.78	56.00	17.00	521.00	61.10	55.40	23.70	Silty-clay-loam
Shiraz, Fars, Iran	2020	8.00	0.93	0.74	1.02	490.00	36.00	43.20	20.80	Clay-loam
	2021	7.80	2.01	0.76	4.40	264.00	36.00	43.20	20.80	Clay-loam
Hamedan, Iran	2020	7.93	6.14	0.45	47.60	499.00	15.50	27.50	53.00	Silty-loam
	2021	7.90	6.00	0.42	47.65	498.00	15.50	27.50	53.00	Silty-loam

### Data collection

After the field evaluations, the experimental genotypes were transferred to the quality control laboratory of the SBSI for the evaluation of their quality traits. The roots were washed and a pulp sample was prepared using an automatic machine. The sample was then stored in a freezer at − 18 °C. In due time, 26 g of the frozen samples were taken and mixed with 177 ml of lead (II) hydroxide acetate for three minutes in a mixer. The resulting solution was then passed through a sieve to obtain a clear liquid. This liquid was used in the Betalyser device (an automatic system for the analysis of sugar beet quality) to measure the SC, N, Na^+^, and K^+^ elements^29^. To calculate the WSY for each genotype, the molasses sugar and the white sugar content were estimated using Eqs. (1) and (2)^30^. These values, along with the RY of each experimental genotype, were then used in Eq. (3) to obtain the WSY^28^. However, all international, national and institutional guidelines have been taken into account in various stages of experiments.

1 $$MS=0.0343\left({K}^{+}+{Na}^{+}\right)+0.094\left(alpha \; amino \; N\right)-0.31$$

2 $$WSC=SC-(MS+0.6)$$

3 $$WSY=WSC\times RY$$

where MS is molasses sugar (%), K^+^ is potassium (meq.100 g^−1^), Na^+^ is sodium (meq.100 g^−1^), *alpha-amino-*N is nitrogen (meq.100 g^−1^), WSC is white sugar content (%), SC is sugar content (%), WSY is white sugar yield (t ha^−1^) and RY is root yield (t ha^−1^).

### Statistical analysis

Before undertaking any form of analysis, first was applied the Grubbs test^40^ to the data, operating under the assumption of normality. As well, the Bartlett test^41^ was employed to check the homogeneity of the variance of experimental errors across various years and locations. After verifying the uniformity of the variance of these errors, the combined analysis of variance was carried out. This analysis was performed on a random year effect, while the location and genotype effects were assumed to be fixed. The analysis was applied to the RY and WSY using Eq. (4)^42^.

4 $${Y}_{ijkl}=\mu +{Y}_{i}+{L}_{j}+{YL}_{ij}+{B}_{\left(ij\right)k}+{T}_{l}+{YT}_{il}+{LT}_{jl}+{YLT}_{ijl}+{BT}_{\left(ij\right)kl}$$

where ${Y}_{ijkl}$ is the response measured on the ${ijkl}{th}$ experimental unit (plot), $\mu$ is the overall mean, ${Y}_{i}$ is the effect of the ${i}{th}$ year, ${L}_{j}$ is the effect of the ${j}{th}$ location, ${YL}_{ij}$ is the interaction effect of the ${i}{th}$ level of Y with the ${j}{th}$ level of L, ${B}_{\left(ij\right)k}$ is the effect of the ${j}{th}$ block within the ${i}{th}$ location, ${T}_{l}$ is the effect of the ${l}{th}$ treatment, ${YT}_{il}$ is the interaction effect of the ${i}{th}$ level of Y with the ${l}{th}$ level of T, ${LT}_{jl}$ is the interaction effect of the ${j}{th}$ level of L with the ${l}{th}$ level of T, ${YLT}_{ijl}$ is the interaction effect of the ${i}{th}$ level of Y with the ${j}{th}$ level of L and the ${l}{th}$ level of T, and ${BT}_{\left(ij\right)kl}$ is experimental error.

To integrate the features of AMMI and BLUP models, the initial step involved computing the scores of these models for each genotype that was tested. These scores were then combined using the WAASB index, as per Eq. (5), resulting in a unified score that included elements from both the AMMI and BLUP modelst>.

5 $${{\text{WAAS}}B}_{{\text{i}}}=\frac{\sum_{{\text{k}}=1}^{{\text{P}}} \left|{{\text{IPCA}}}_{{\text{ik}}}\times {{\text{EP}}}_{{\text{k}}}\right|}{\sum_{{\text{k}}=1}^{{\text{P}}} {{\text{EP}}}_{{\text{k}}}}$$

where (Eq. 5) ${{\text{WAASB}}}_{{\text{i}}}$ is the weighted average of absolute scores of the ${i}{th}$ genotype or environment; ${{\text{IPCA}}}_{{\text{ik}}}$ is the absolute score of the ${i}{th}$ genotype or environment in the ${k}{th}$ interaction principal component (IPC); and ${{\text{EP}}}_{{\text{k}}}$ is the magnitude of the variance explained by the ${k}{th}$ IPC. Given that the attainment of high-performance, stable genotypes is a primary objective, the individual contributions of GEI were examined using a BLUP matrix. Following this analysis, the WAASBY index, which measures both yield average and stability, was calculated for each genotype as per Eq. (6)^37^.

6 $${{\text{WAAS}}BY}_{{\text{i}}}=\frac{\left({rG}_{g}\times {\theta }_{Y}\right)+({rW}_{g}\times {\theta }_{S})}{{\theta }_{Y}+{\theta }_{S}}$$

where ${{\text{WAAS}}BY}_{{\text{i}}}$ is the superiority index with different weights between yield and stability for the ${g}{th}$ genotype; ${\theta }_{Y}$ and ${\theta }_{S}$ are the weights for yield and stability, respectively; ${rG}_{g}$ and ${rW}_{g}$ are the rescaled values of the ${g}{th}$ genotype for yield and WAASB, respectively. The correlation analysis of key traits was performed using the Pearson method. The first step in examining the GYT involved standardizing the original data. This was done following Eq. (7), as suggested by Yan and Frégeau-Reid^24^.

7 $${P}_{ij}=\frac{{T}_{ij}-{\overline{T} }_{j}}{{S}_{j}}$$

where ${P}_{ij}$ is the standardized value of genotype i for trait or yield-trait combination j in the standardized table, ${T}_{ij}$ is the original value of genotype i for trait or yield-trait combination j, ${\overline{T} }_{j}$ is the mean across genotypes for trait or yield-trait combination j, and ${S}_{j}$ is the standard deviation for yield-trait combination j. The biplot representing the GYT was constructed using the first and second principal components (PCs) derived from the singular value decomposition of the standardized data. In this model, the singular value decomposition breaks down the GYT data into genotype eigenvalues, yield-trait combination eigenvalues, and singular values. This process is based on Eq. (8) as proposed by Yan and Frégeau-Reid^24^.

8 $${{\text{P}}}_{{\text{ij}}}=\left(d{\lambda }_{1}^{\alpha }{\zeta }_{{\text{i}}1}\right)\left({\lambda }_{1}^{1-\alpha }{\tau }_{1{\text{j}}}/{\text{d}}\right)+\left(d{\lambda }_{2}^{\alpha }{\zeta }_{{\text{i}}2}\right)\left({\lambda }_{2}^{1-\alpha }{\tau }_{2{\text{j}}}/{\text{d}}\right)+{\varepsilon }_{{\text{ij}}}$$

where ${\zeta }_{{\text{i}}1}$ and ${\zeta }_{{\text{i}}2}$ are the eigenvalues for PC1 and PC2, respectively, for genotype i; ${\tau }_{1{\text{j}}}$ and ${\tau }_{2{\text{j}}}$ are the eigenvalues for PC1 and PC2, respectively for trait j, and ${\varepsilon }_{{\text{ij}}}$ is the residual from fitting the PC1 and PC2 for genotype i on trait j; ${\uplambda }_{1}$ and ${\uplambda }_{1}$ are the singular values for PC1 and PC2, respectively. α is the singular value partitioning factor. The GYT biplot is created by plotting $d{\lambda }_{1}^{\alpha }{\zeta }_{{\text{i}}1}$ against $d{\lambda }_{2}^{\alpha }{\zeta }_{{\text{i}}2}$ for genotypes, and ${\lambda }_{1}^{1-\alpha }{\tau }_{1{\text{j}}}/{\text{d}}$ against ${\lambda }_{2}^{1-\alpha }{\tau }_{2{\text{j}}}/{\text{d}}$ for yield-trait combinations. This analysis focuses on four main patterns: (1) Exploring the associations between different yield-trait combinations, (2) Identifying the best genotype for each yield-trait combination, (3) Ranking genotypes based on a superiority index and evaluating their strengths and weaknesses, and (4) Ranking genotypes based on an ideal hypothetical genotype.

To identify the most superior sugar beet genotypes, a comprehensive evaluation of key traits such as RY, SC, N, Na^+^, and K^+^ is necessary. In order to assess the stability of these traits simultaneously, the MTSI index was calculated using Eq. (9)^37^.

9 $${MTSI}_{i}={\left[\sum_{j=1}^{f} {\left({\gamma }_{ij}-{\gamma }_{j}\right)}^{2}\right]}^{0.5}$$

where ${MTSI}_{i}$ is the multi-trait stability index of the genotype *i*, ${\gamma }_{ij}$ is the score of the genotype *i* in the factor *j*, and ${\gamma }_{j}$ is the score of the ideal genotype in the factor *j*. The scores were calculated using factor analysis for genotypes and traits. Finally, stable genotypes were selected based on positive selection differentials for traits intended to increase and negative selection differentials for traits intended to decrease.

## Results and discussion

### Combined analysis of variance

The non-significance of the G statistic in the Grubbs test was determined based on the normality of the experimental data for RY and WSY. The non-significant chi-square values obtained from Bartlett test for RY and WSY further confirmed the uniformity of experimental error variance across 10 environments (five regions in two years). After confirming these hypotheses, a combined analysis of variance was conducted to describe the main effects and quantify the interactions between different sources of variations. The mean of square of the main effects of year and genotype at the 1% probability level, and location at the 5% probability level, showed significant differences for both RY and WSY. The interaction effects of year-location, genotype-year, and genotype-year-location at the 1% probability level, as well as genotype-location at the 5% probability level, resulted in a significant difference in RY. These interaction effects, except for the year-genotype interaction, had a similar impact on WSY as they did on RY (Table 5). The significant variations observed in experimental years, locations, and genotypes can be attributed to changes in environmental conditions and the genetic makeup of plants, which vary from one environment to another. This leads to variations in RY and WSY among different experimental genotypes in different environments. These findings confirm the inevitability of various interactions in agricultural research^26,43^. The GEI primarily indicates that genotypes respond differently to various locations, emphasizing the need to evaluate genotypes in diverse environments. Based on the obtained results from the experiment conducted by Sadeghzadeh Hemayati et al.^9^ the environment and its interaction with the genetic structure of different genotypes played a significant role in the phenotypic expression of WSY in sugar beet genotypes. This resulted in different responses in terms of WSY based on the conditions of different environments. Similarly, the study conducted by Saremirad and Taleghani^13^ indicated that GEI outweighs the quantitative and qualitative characteristics of sugar yield in sugar beet hybrids. Therefore, this interaction should be considered when breeding new hybrids, as it allows for decisions regarding breeding for general or specific adaptation, depending on the yield stability in different environmental conditions. In order to better understand and reveal the GEI, multivariate statistical methods can be more useful. These methods can provide insights into the complex relationships between genotypes and environments, allowing for a more comprehensive understanding of the factors influencing crop yield and the development of cultivars with stability and adaptability to target environments.

**Table 5:** Combined analysis of variance of root and white sugar yields in experimental sugar beet genotypes.

Source of variation	df	Root yield		White sugar yield	
		Sum of square	Mean of square	Sum of square	Mean of square
Year	1	17,160.54	17,160.54**	184.86	184.86**
Location	4	130,304.80	32,576.20*	3129.38	782.34*
Year × locality	4	15,725.22	3931.30**	324.82	81.20**
Error 1	30	3256.01	108.53	190.16	6.34
Genotype	19	5760.54	303.19**	270.29	14.23**
Year × genotype	19	2037.18	107.22**	35.70	1.88^ ns^
Locality × genotype	76	12,582.24	165.56*	286.76	3.77*
Year × locality × genotype	76	7932.13	104.37**	180.21	2.37**
Error 2	570	28,409.00	49.84	905.95	1.59

**: 1% probability level of significance; *: 5% probability level of significance; ns: non-significant.

### Integration of AMMI and BLUP models

To enhance the reliability of analysis results in multi-environment experiments and gain a more precise understanding of GEI, was conducted an analysis of RY and WSY data using a combination of AMMI and BLUP models. The RY and WSY of the experimental genotypes were significantly influenced by the effects of environment, genotype, and GEI, according to the results of the analysis of variance based on the AMMI model. These factors accounted for 66.41%, 2.41%, and 9.18% of the variations in RY and 60.54%, 4.50%, and 8.36% of the variations in WSY, respectively (Table 6). Thus, the phenotypic expression of RY and WSY is determined by the genotype's genetic makeup, the field's environmental factors, and how these factors interact. Due to the polygenic nature of these qualities, environmental factors, genomic regions with linked genes, and quantitative trait loci all continuously affect them^44^. The genes that affect yield and its components are highly sensitive to environmental conditions and exhibit an interaction between quantitative trait loci and the environment. This interaction can either facilitate or limit the response to selection^45^. Therefore, it is crucial for breeding programs to consider and manage these effects properly^46^.

**Table 6:** An AMMI-based analysis of variance, likelihood ratio test, and estimation of genetic variance components for root and white sugar yields in experimental sugar beet genotypes.

Source of variation	df	Root yield			White sugar yield		
		Mean of square	Pro.	Acc.	Mean of square	Pro.	Acc.
Environment	9	18,132.28**	66.41	66.41	404.34**	60.54	60.54
Replication (environment)	30	108.53	–	–	6.34	–	–
Genotype	19	303.19**	2.34	70.08	14.23**	4.50	68.20
Genotype × environment	171	131.88**	9.18	79.26	2.94**	8.36	76.57
PC1	27	241.30**	28.90	28.90	5.18**	27.80	27.80
PC2	25	192.93**	21.40	50.30	4.51**	22.40	50.30
PC3	23	183.30**	18.70	69.00	3.48**	15.90	66.20
PC4	21	121.26**	11.30	80.30	2.55*	10.70	76.90
PC5	19	109.65**	9.20	89.50	2.76*	10.40	87.30
PC6	17	59.30^ns^	4.50	94.00	1.76^ns^	5.90	93.30
PC7	15	40.90^ns^	2.70	96.70	1.11^ns^	3.30	96.60
PC8	13	43.51^ns^	2.50	99.20	0.84^ns^	2.20	98.80
PC9	11	16.34^ns^	0.80	100.00	0.56^ns^	1.20	100.00
Residuals	570	49.84	–	–	1.59	–	–
Total	970	253.32	–	–	6.20	–	–
Likelihood ratio test							
Environment		49.84**	66.78	66.78	1.59**	71.95	71.95
Genotype		4.30**	5.74	72.52	0.28**	12.77	84.72
Genotype × environment		20.51**	27.48	100	0.34**	15.28	100
Genetic variance component							
${\updelta }_{{\text{P}}}^{2}$		74.63			2.21		
${{\text{h}}}_{{\text{mg}}}^{2}$		0.56			0.79		
${{\text{R}}}^{2}$ genotype × environment		0.27			0.15		
Accuracy		0.75			0.89		

**: 1% probability level of significance; *: 5% probability level of significance; ns: non-significant; Pro: proportion variance; Acc: accumulated variance.

The analysis of the multiplicative interaction on RY was performed using principal components analysis. The first five IPCs were found to have significant differences at the 1% probability level and collectively accounted for 89.50% of the GEI sum of square. The contribution of each IPC was as follows 28.90%, 21.40%, 18.70%, 11.30%, and 9.20%, respectively. A similar analysis was conducted WSY, and the first, second, and third IPCs were found to be significantly different at the 1% probability level, while the fourth and fifth IPCs were significantly different at the 5% probability level. These IPCs explained 87.30% of the GEI variations, with contributions of 27.80%, 22.40%, 15.90%, 10.70%, and 10.40%, respectively. Other studies have also investigated the GEI using different models. Omrani et al.^47^ found that the first four IPCs explained 83% of the GEI variations. Fathi et al.^48^ estimated the contribution of the first and second IPCs to be 49.10% and 22.50%, respectively, accounting for 71.60% of the GEI variations. Mostafavi and Saremirad^49^ reported that the first IPC explained about 63% of the data variation. Rajabi et al.^8^ found that the first six IPCs of the GEI had a significant effect and explained 98.80% of the total variations. Sadeghzadeh Hemayati et al.^9^ identified seven significant IPCs that explained 95.50% of the variations related to the GEI. Based on the results of these studies and the present study, it is clear that the first two IPCs of the GEI alone may not fully explain the variation. Therefore, it is recommended to combine the AMMI model power with the BLUP model prediction accuracy for a more comprehensive interpretation.

The likelihood ratio test (LRT) confirmed the results of the AMMI analysis. The results indicated that the effects of genotype, environment, and GEI were significant at the 1% probability level for both RY and WSY. The significant effect of GEI suggests that the experimental genotypes respond to changes in environmental conditions. In other words, the strengths and weaknesses of each experimental genotype are determined by the environmental conditions. In such situations, using BLUP analysis to estimate genetic variance components can provide more reliable and accurate results^37^. Consequently, the REML/BLUP method was used to estimate the values of genetic variance components for both studied traits (Table 6). The environmental variance accounted for the highest proportion of phenotypic variance in RY (66.78%) and WSY (71.95%). The GEI variance ranked second, explaining 27.48% of the phenotypic variation in RY and 15.28% in WSY. The genotypic variance ranked third, explaining 5.74% of the phenotypic variation in RY and 12.77% in WSY. The genotypic variance had the lowest share of phenotypic variance in both studied traits. This suggests that environmental factors have a greater impact on the expression and variation of these traits compared to genetic factors. In other words, the influence of genes in creating diversity among genotypes is low. Therefore, it can be concluded that the genotypes are strongly influenced by environmental conditions in terms of RY and WSY. Consequently, the efficiency of selection decreases, and the identification and selection of desirable genotypes become less accurate^50^. The low variance of the GEI compared to the environmental variance indicates that the genetic variation has a limited effect on the phenotypic expression of RY and WSY in different sugar beet genotypes. This leads to low yields fluctuating from one environment to another. When the GEI variance is low compared to the environmental variance, it suggests that genetic factors have a limited influence on the phenotypic expression of desired traits^51^. This finding is important for breeders in terms of trait selection. The study by Taleghani et al.^17^ on sugar beet winter cultivation confirmed that the environment has the greatest effect on WSY, explaining 71.50% of the data variations. The genotype accounted for 9.80% of the justified variance, while the GEI accounted for 7.20% of the data variations. In the study by Basafa and Taherian^52^, the variance justified by GEI was 7.84%. Mostafavi and Saremirad^49^ reported that the environment, genotype, and GEI justified variances of 17.69%, 32.79%, and 17.90% of the total sum of squares, respectively. Their study also found that genotype had the greatest variation in yield, indicating a high diversity of genotypes.

Heritability plays a crucial role in breeding program, helping plant breeders select plants with desirable traits and develop effective breeding strategies^37,53^. Breeders can choose which traits to target for improvement and what breeding techniques to employ by studying the heritability of traits^54,55^. The degree of agreement between a trait's genotypic and phenotypic values is measured statistically as heritability. It measures the proportion of genetic influences that are responsible for the observed variation in a trait within a population. An estimate of the trait's heritability, with a possible range of 0 to 1, is made. Low heritability is defined as a heredity value less than 0.2, medium heritability is defined as a heritability value between 0.2 and 0.5, and high heritability is defined as a heritability value larger than 0.5. In the case of RY and WSY, the results obtained showed moderate to high heritability. This means that the phenotype of these traits is a good indicator of the underlying genotype and selection based on phenotype is likely to be effective. The genotypic correlation between environments was found to be low. A low correlation suggests difficulties in selecting superior stable genotypes across different environments. In the context of RY and WSY, the low correlation indicates that the genetic effects of these traits were not consistent across different environments. Therefore, accurate information and details are needed to select superior genotypes in such cases^38^. Selection accuracy is another important metric in breeding programs. It measures the correlation between observed and predicted values^37^. In the case of the mentioned traits, RY and WSY, the selection accuracy was high, with values of 0.75 and 0.89, respectively (Table 6). These high selection accuracy values indicate the reliability of the model in identifying superior genotypes for these traits.

**Figure 1:**
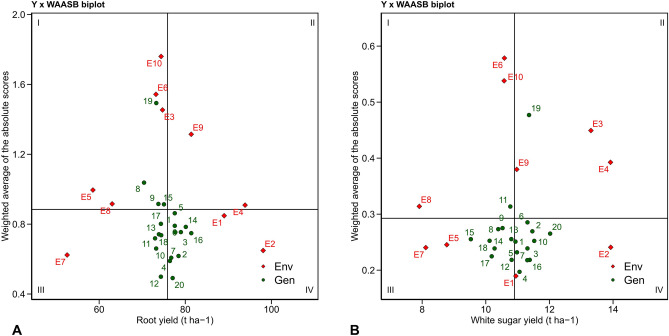
Biplot analysis of root yield (**A**) and white sugar yield (**B**) of sugar beet genotypes, evaluated by weighted average absolute scores of the best linear unbiased predictions (WAASB).

Although most genotypes variation can be explained by the first two IPCs obtained from the AMMI model, it is possible that some genotypes may require more IPCs. Therefore, the WAASB index can be used as a quantitative measure of stability that considers these variations. Figure 1 depicts a biplot where performance traits, such as RY and WSY, are represented on the horizontal axis, while WAASB index values are shown on the vertical axis. In this biplot, the vertical line in the middle represents the total yield average in the experimental environments. Genotypes and environments to the right of this line have a yield value higher than the total average, whereas those to the left have a lower yield value. The horizontal axis in the middle of the biplot represents the average area of the WAASB index. The intersection of this axis with the vertical axis (yield average) divides the biplot into four quadrants. Based on their stability in various environments, genotypes may be categorized into the biplot's several quadrants. In particular, the first quadrants of the RY biplot (genotypes 19, 8, 9, and 15, and environments E10, E6, E3, and E5) and the WSY biplot (genotype 11 and environments E6, E10, and E8) had genotypes and environments with high WAASB values and yields that were below the total average. This reveals both their very variable and unstable character and their yield value, which is below average. In other words, these genotypes and environments played a significant role in GEI, and they are not recommended for cultivation. On the other hand, the environments E9 and E4 in the second quadrant of the RY biplot and genotype 19 and environments E3, E4, and E9 in the same quadrant of the WSY biplot had high WAASB values and yields higher than the total average. These genotypes have good recognition ability and, when the environmental conditions are favorable, can achieve very high yield values. Therefore, they can be recommended for cultivation in areas with ideal conditions for sugar beet growth and development. In the RY biplot, genotypes 12, 10, 11, 18, 13, and 17, as well as environment E7, were placed in the third quadrant. In the WSY biplot, genotypes 12, 17, 18, 14, 15, 13, 8, and 9, along with environments E5 and E7, were also placed in the third quadrant. These genotypes and environments exhibited a lower WAASB index, indicating their stability and lack of influence from environmental conditions. Additionally, these genotypes showed low yield values. Essentially, the mentioned genotypes had low yield and high stability in this quadrant. On the other hand, the environments in this quadrant had low efficiency and a lower separation capability due to having the lowest WAASB values among all the environments. In contrast, genotypes 20, 4, 7, 2, 16, 3, 6, 1, 14, and 15, along with environments E2 and E1, were located in the fourth quadrant of the RY biplot. Similarly, genotypes 4, 16, 3, 7, 5, 1, 10, 20, 2, and 6, along with environments E1 and E2, were located in the fourth quadrant of the WSY biplot. These genotypes and environments had low WAASB index values and higher yield values compared to the total average. Genotypes in this biplot quadrant are considered stable and optimal in terms of yield because they have minimal influence on environmental conditions while achieving proper yields. The environments placed in the fourth quadrant had high production capacity and low WAASB values among the 10 experimental environments. The WAASB index is used to identify genotypes with optimal and stable yield in various plants, including wheat^56^, soybeans^57^, lentils^58^, rice^59^, corn ^60^, and sugar beet^9,11^. Unlike the AMMI model, which considers only the first IPC, the WAASB index expresses stability based on all scores of the IPCs. Therefore, WAASB considers the total GEI variance in identifying stable genotypes. In situations where the number of significant IPCs is high and the IPC1 cannot explain most of the GEI, it is suggested to use the WAASB index. This index fully applies the variance of GEI in identifying stable genotypes. The BLUP-based mixed model, which incorporates the WAASB index, has been shown to perform more accurately than the fixed-effects AMMI model in identifying stable genotypes^60–63^. The WAASB index has been successfully used, and provides useful results in identifying genotypes with optimal and stable yield.

**Figure 2:**
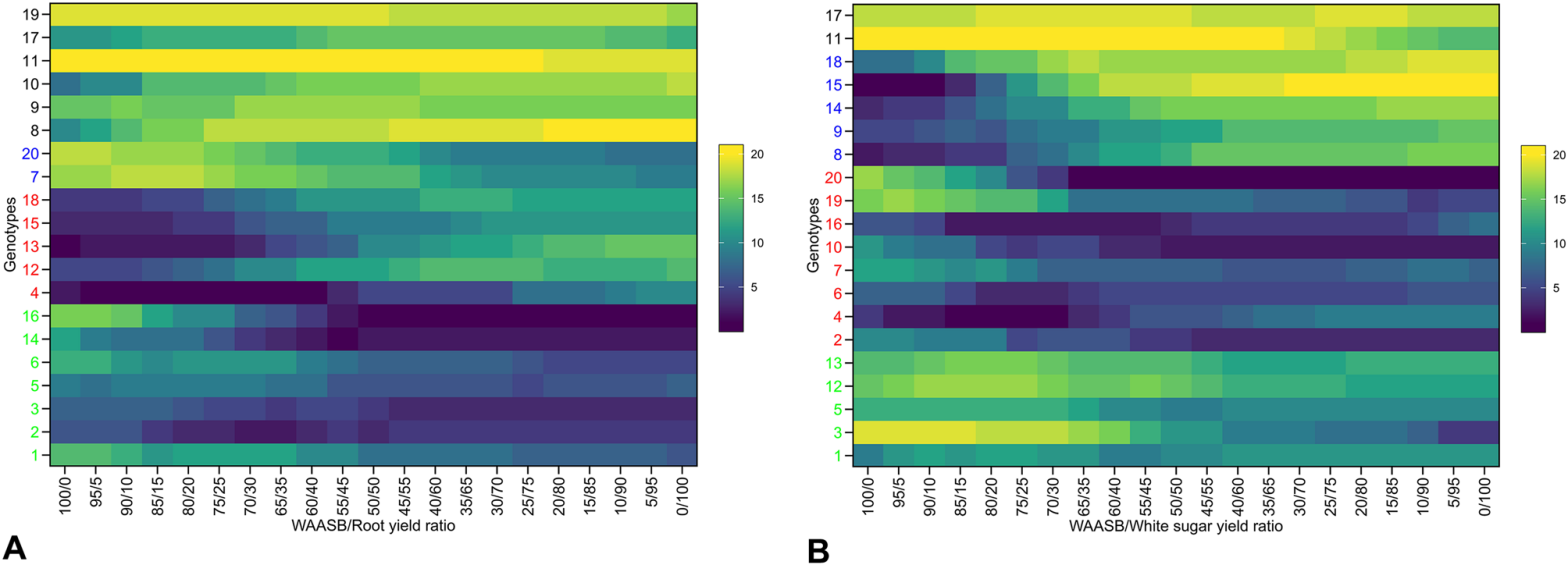
Ranking of sugar beet genotypes based on varying importance of stability and root yield (**A**)/white sugar yield (**B**).

**Figure 3:**
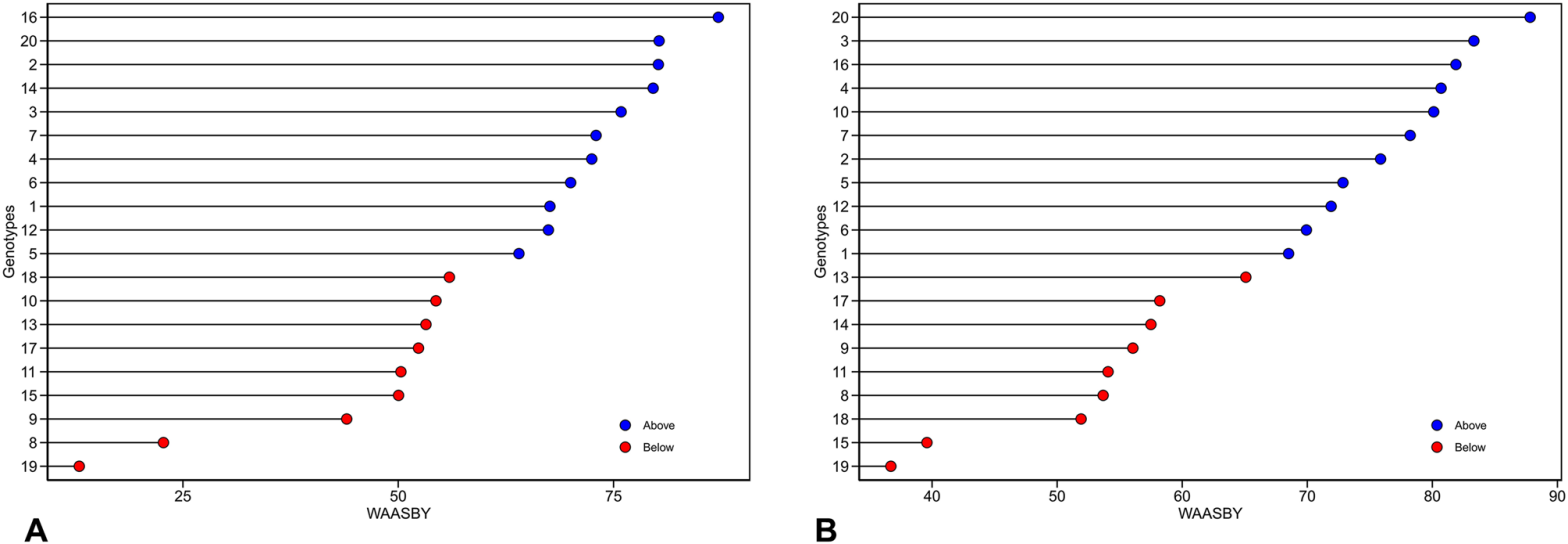
Calculation of WAASBY in sugar beet genotypes, considering equal weights of 50 for root yield (**A**)/white sugar yield (**B**), and WAASB.

To evaluate yield and stability with greater precision, experimental genotypes were ranked based on their WAASBY index scores, as depicted in Fig. 2. These scores, which take into account various ratios of WAASB index to yield, were illustrated in a heatmap for RY (Fig. 2A) and WSY (Fig. 2B). The ranking of genotypes via the WAASBY index can fluctuate depending on the ratio of the WAASB index to yield. The first component (on the left side of the diagonal line) of the WAASB index to yield ratio (WAASB/RY or WSY) pertains to environmental stability, while the second component (on the right side of the diagonal line) relates to yield (RY or WSY). Thus, in the genotype ranking, a 0/100 ratio corresponds to stability, while the same ratio is also assigned to yield. Indeed, moving one unit from left to right in this plot decreases the environmental stability component by five percent and increases the yield component, such that at the end, genotypes are ranked solely based on yield (0/100). Based on the WAASBY index scores, the experimental genotypes were categorized into four groups. The first group (green) includes genotypes 1, 2, 3, 5, 6, 14, and 16 for RY, and genotypes 1, 3, 5, 12, and 13 for WSY. These genotypes were identified as stable with optimal yield. The second group (red), with poor yield values and instability, includes genotypes 4, 12, 13, 15, and 18 for RY, and genotypes 2, 4, 6, 7, 10, 16, 19, and 20 for WSY. The third group (blue), which includes genotypes 7 and 20 for RY and genotypes 8, 9, 14, 15, and 18 for WSY, demonstrated high yield values but instability. The fourth group (black) contains genotypes 8, 9, 10, 11, 17, and 19 for RY, and genotypes 11 and 17 for WSY, which showed poor yield values but were stable otherwise. In multi-environment experiments, the WAASBY index has proven to be a valuable selection index for both stability and yield, as it allows breeders to select genotypes with varying ratios of stability and yield based on their breeding program objectives. Figure 3 is particularly useful for selecting genotypes with an equal ratio. This figure allows for the simultaneous ranking and selection of experimental genotypes based on a 50:50 ratio for the WAASB index and RY (Fig. 3A) and WSY (Fig. 3B). Blue circles indicate a WAASBY index above the total average, while red circles indicate a WAASBY index below the total average. In summary, for RY, 11 genotypes, including genotypes 16, 20, 2, 14, 3, 7, 4, 6, 1, 12, and 5, had a higher than WAASBY index. Specifically, genotype 16 and thereafter genotypes 20, 2, and 14 had relatively higher WAASBY index values compared to other genotypes. For WSY, the results were largely similar to RY, with only the ranking of genotypes differing. On this trait, 11 genotypes, including genotypes 20, 3, 16, 4, 10, 7, 2, 5, 12, 6, and 1, had a higher than WAASBY index. In this case, genotype 20, followed by genotypes 3, 16, 4, and 10 had relatively higher WAASBY index values compared to other genotypes. According to these results, genotypes 16, 20, 2, and 14 for RY values and yield stability, and genotypes 20, 3, 16, 4, and 10 for WSY values and yield stability were identified as suitable genotypes. Sugar beet, a critical sugar plant that provides about 30% (approximately 42 million tons) of the world sugar needs (FAO, 2018), is of considerable importance, especially in light of climate change concerns and the need to transition from fossil fuel consumption to renewable fuels. Developing genotypes with high and stable performance under varying environmental conditions is a significant solution^8,9,11^. In light of this, the WAASBY index, a quantitative stability measure, is used to balance yield average and stability. This index allows for the selection of genotypes that have both high yield and yield stability^11,64,65^.

### Exploring the correlation and graphical analysis of genotype by yield*trait (GYT)

**Figure 4:**
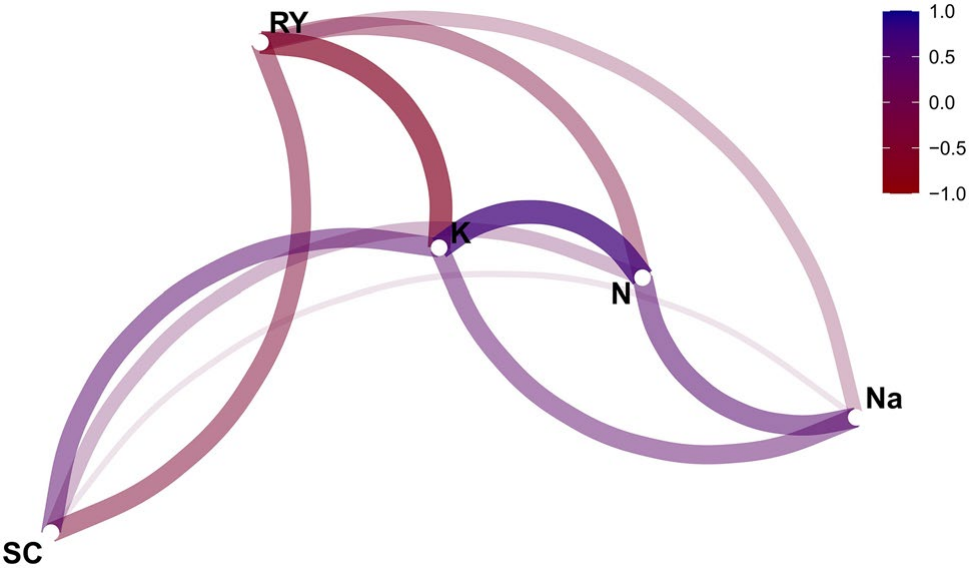
Pearson correlation analysis among key traits of sugar beet genotypes.

The Pearson correlation coefficient was utilized to determine the degree of association between various traits. The values of this coefficient range from − 1 to 1. A correlation coefficient of 1 indicates a complete direct association between two traits, meaning that if one trait increases or decreases, the other will also increase or decrease proportionally. As the coefficient approaches zero, the direct association weakens, resulting in no linear association between the two traits. Conversely, as the coefficient approaches − 1, the inverse association between the two traits intensifies, leading to a complete inverse association when the correlation coefficient is − 1. This implies that if one trait increases, the other decreases and vice versa. The results of Pearson's correlation of various studied traits of experimental sugar beet genotypes are depicted in Fig. 4. The highest positive correlation between N and K^+^ was observed at a rate of 0.71. This high degree of correlation between the two traits suggests that an increase in the amount of N also results in an increase in the amount of K^+^. The traits of Na with N (r = 0.49), SC with K^+^ (r = 0.45), and Na^+^ with K^+^ (r = 0.41) exhibited a weak positive correlation. RY exhibited a negative correlation with all traits, including the SC, Na^+^, K^+^, and N. However, the highest negative correlation between RY and K^+^ (r = − 0.65) was observed, indicating that K^+^ decreases with an increase in RY. There was a weak negative correlation between RY with SC (r = − 0.44), RY with N (r = − 0.37), and RY with Na^+^ (r = − 0.19). A similar relationship between RY and SC has been reported previously^66,67^. The correlation of SC with Na was estimated to be negative but non-significant. The associations between the key traits of sugar beet can pose complications in the process of improving these traits. Therefore, breeders must strike a balance while managing the associations imposed by negative correlations between key traits. In this context, the GYT graphical method proves to be comprehensive and efficient^24^.

To comprehend the impact of genetic factors on crop productivity, was employed a method known as GYT graphical analysis. The results revealed that the first and second PCs accounted for 50.53% and 34.96% of the total variance in RY data, respectively, summing up to 85.49%. Given that these two PCs explain over 70% of the data variance, this affirms the high validity of the biplots obtained from this study in elucidating the variations in GYT^68^. However, if the sum of the first two PCs does not account for the majority of the data variation, it indicates the data complex nature^69^. Nevertheless, this does not invalidate the biplot^27^. Research by Shojaei et al.^70^ demonstrated that about 50% of the variation in GYT is explained by the first two PCs. In an experiment conducted by Faheem et al.^36^, they estimated that the sum of the first two PCs was close to 85%, with the PC1 accounting for approximately 74% and the PC2 for around 11% of the total data variations.

**Figure 5:**
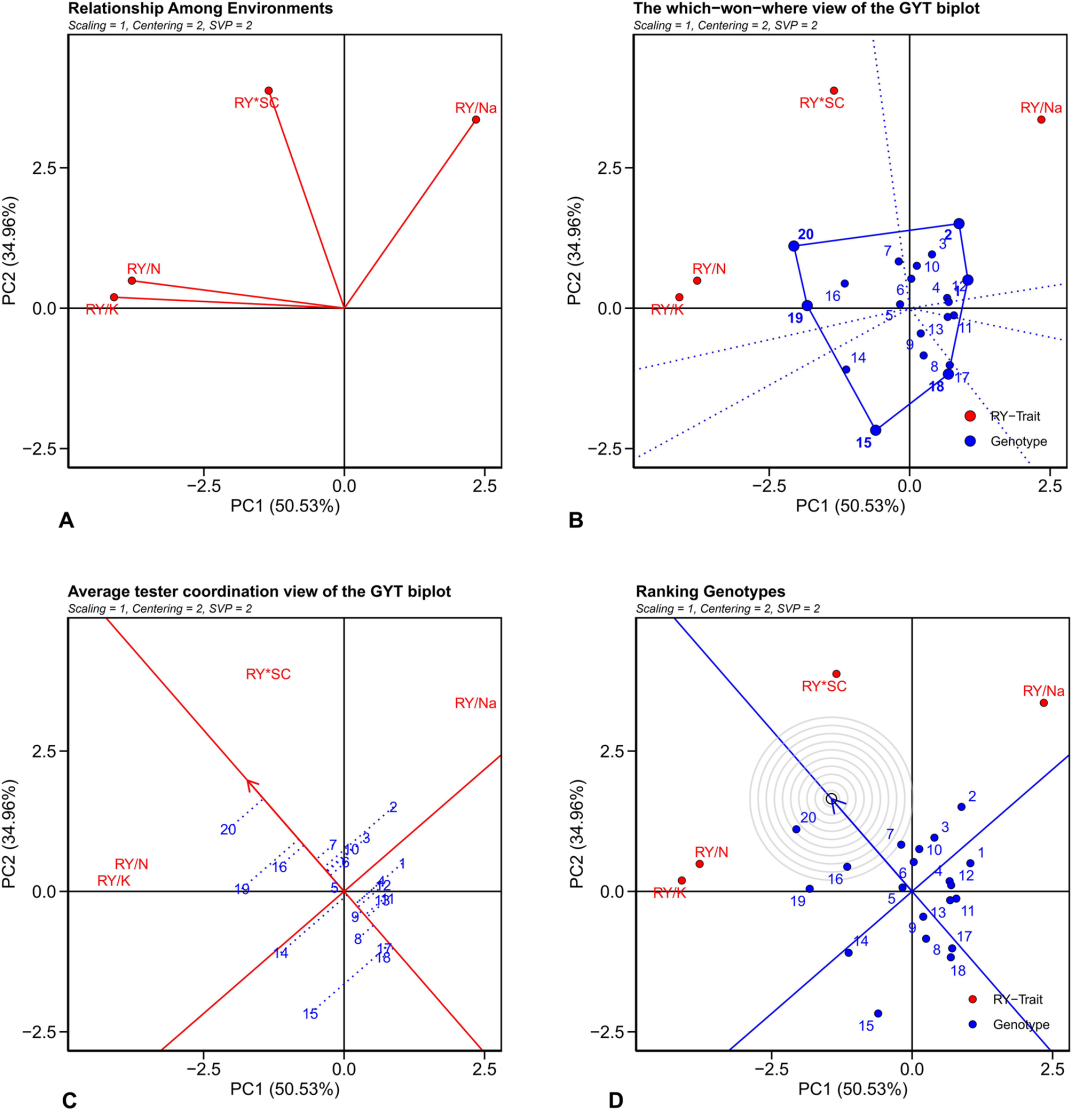
Different views of the genotype by root yield*trait biplot for yield-trait associations (**A**), highlighting genotypes with outstanding profiles (**B**), ranking genotypes based on their overall superiority and their strengths and weaknesses (**C**) and ranking genotypes based on the ideal genotype (**D**).

The quality and yield of sugar from sugar beet roots are influenced by impurities such as N, Na^+^, and K^+^. The N content, which is a combination of soluble amino acids and amide groups, plays a significant role in sugar extraction efficiency^71^. Although high N levels can impede sugar extraction, it is a vital factor in the storage life and quality of sugar beet. Hence, managing N levels, which can be affected by temperature, storage duration, and beet genetic diversity, is critical^72^. Na^+^ and K^+^ in sugar beet roots are considered molasses substances. They increase the solubility of sucrose, reducing its crystallization, which in turn decreases the quality of sugar beet and the efficiency of sugar extraction^73–76^. A high concentration of these elements can limit their use in certain applications due to their impact on the quality of molasses. Therefore, it is essential to design corrective programs that increase RY while also reducing the concentration of impurities. Studying the correlation between yield-trait combinations can identify the association among traits, aiding future experiments in creating new genotypes. In this context, the acute angle between the majority of vectors in a graph indicates a positive correlation among yield-trait combinations, mainly due to yield being a PC in these combinations^24^. This high correlation suggests a strong relationship among the rank of genotypes based on these combinations. The biplot (Fig. 5A) reveals a high positive correlation between RY/N and RY/K^+^. This indicates that reducing the content of one of these traits will likely result in a reduction in the other. There is also a weak positive correlation among these compounds and the combination of RY*SC. However, they show a negative correlation with RY/Na^+^. There is a moderate positive correlation between RY*SC and RY/Na^+^. The positive correlation between RY*SC and other compounds suggests that it is possible to increase the SC by reducing the N, Na^+^, and K^+^ in a genotype. However, the negative correlation among RY/N, RY/K^+^, and RY/Na^+^ complicates the breeding process to reduce all three impurities, necessitating detailed planning (Fig. 5A).

In Fig. 5B, a polygon is visible, formed by connecting genotypes 2, 12, 18, 15, 19, and 20, which are furthest from the coordinate origin. Perpendicular lines extending from the coordinate origin to the polygon sides helped determine the grouping of GYT combinations. When yield-trait combinations are positioned with genotypes at their apex, it suggests these genotypes yield well in terms of those combinations. In other words, these are the best genotypes with respect to yield-traits. genotypes 2 and 12 excel in the RY/Na^+^. For the combinations of RY*SC, RY/N, and RY/K^+^, genotypes 20, 19, and 16 respectively, are the best genotypes (Fig. 5B). Genotypes located in sections devoid of combinations are not desirable for any combination and are considered weaker genotypes. The polygonal biplot also helped group yield-trait combinations into two groups. The RY/Na^+^ was placed in one group, while RY*SC, RY/N, and RY/K^+^ were grouped together (Fig. 5B).

The genotypes ranking, based on the yield-trait combination, was determined using the average tester coordinate biplot (Fig. 5C) and the superiority index (Table 7). The axis on the biplot, represented by an arrow originating from the coordinate’s origin, illustrates the mean of all yield-trait combinations. The axis perpendicular to this average axis signifies the measurement of the genotypes balance in terms of compounds. From this information, genotype 20 was identified as the best, followed by genotypes 19 and 16, due to their high yield average. On the other hand, genotypes 15, 18, and 17 exhibited the lowest yield average. These biplot results were corroborated by the superiority index. Various studies have been conducted^24,36,70^, including the present one, which concludes that the average tester coordinate biplot is a useful method in the GYT biplot analysis. This method provides valuable information about genotypes. The identification of the ideal genotype is based on the concepts of balance and high yield. Accordingly, the most desirable genotype is one that has the highest yield and maximum balance. Genotypes that are closest to this ideal are deemed superior, while those farthest are considered the undesirable. According to the biplot, genotypes 20, 16, 7, and 19 were identified as the best genotypes due to their minimal distance from the ideal genotype. Conversely, genotypes 15, 18, and 17 were identified as unfavorable genotypes because they were the furthest from the ideal genotype (Fig. 5D).

**Table 7:** Standardized data of genotype by root yield*trait and superiority index for sugar beet genotypes.

Genotype	Root yield × sugar content	Root yield/${{\text{Na}}}^{+}$	Root yield/${{\text{K}}}^{+}$	Root yield/*alpha amino* N	Mean superiority index
1	0.31	0.85	− 1.21	− 0.93	− 0.24
2	1.27	1.69	− 0.84	− 0.74	0.35
3	1.10	0.74	− 0.41	− 0.50	0.23
4	0.44	0.04	− 0.82	− 0.95	− 0.32
5	0.44	− 0.37	− 0.01	0.06	0.03
6	0.76	0.20	− 0.06	− 0.16	0.18
7	0.48	0.99	0.23	0.70	0.60
8	− 1.59	0.13	− 0.23	0.26	− 0.36
9	− 0.88	0.09	− 0.52	0.43	− 0.22
10	0.31	1.20	0.94	− 0.72	0.43
11	− 0.59	0.54	− 0.97	− 0.42	− 0.36
12	− 0.35	0.73	− 0.73	− 0.41	− 0.19
13	− 0.39	0.28	− 0.64	− 0.72	− 0.37
14	− 0.08	− 2.18	1.33	0.13	− 0.20
15	− 1.89	− 2.20	0.89	− 0.11	− 0.82
16	1.17	− 0.62	1.64	0.38	0.64
17	− 1.21	− 0.48	− 1.01	− 0.60	− 0.82
18	− 1.26	− 0.71	− 0.89	− 0.80	− 0.92
19	0.32	− 0.75	1.22	2.77	0.89
20	1.66	− 0.17	2.07	2.34	1.48
Mean	0.00	0.00	0.00	0.00	–
Standard deviation	1.00	1.00	1.00	1.00	–

### Multi-trait stability index (MTSI)

**Figure 6:**
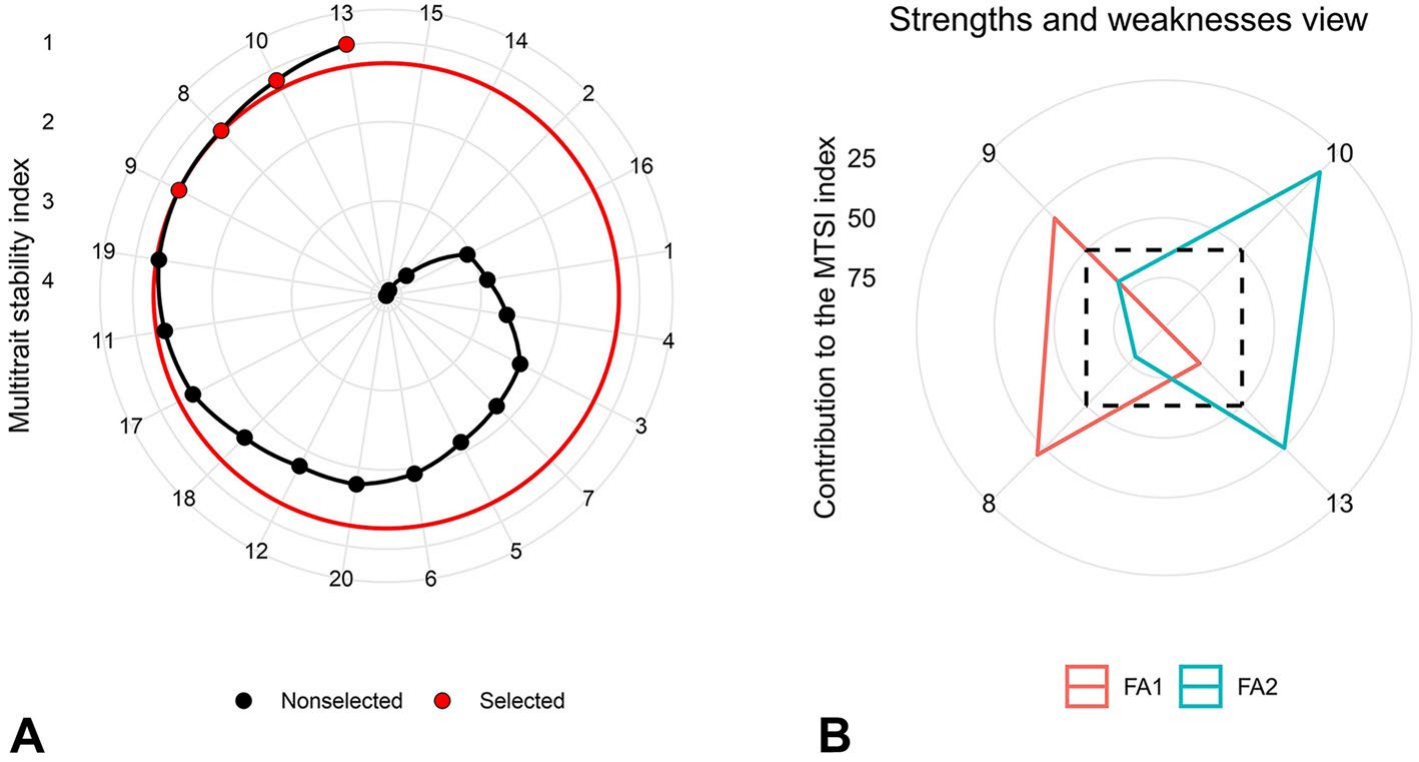
Ranking of genotypes in ascending order based on the multitrait stability index (**A**), and evaluation of selected genotypes in terms of their strengths and weaknesses represented as the ratio of each factor in the calculated mult-itrait stability index (**B**).

Factor analysis was performed using principal component analysis, and the interpretation of the results was carried out after Varimax rotation. Table 8 presents the results of the factor analysis, where factors with eigenvalues greater than one were selected. The variance of each factor was expressed as a percentage, indicating its importance in interpreting the overall variations in the data. In this analysis, two independent factors accounted for a total of 78.24% of the data variations. The first factor explained 41.76% of the total variance and had an eigenvalue of 2.09. It had large positive coefficients for RY and negative coefficients for K^+^ and N. The second factor, with eigenvalues of 1.82, accounted for 36.48% of the variations and included large negative factor coefficients for SC and Na^+^. The MTSI index of the studied genotypes was calculated based on the factor scores of the two mentioned factors. The MTSI index is used to assess the stability of genotypes, with lower values indicating closer proximity to the stable ideal genotype. Conversely, higher values suggest a greater distance from the ideal stable genotype and should not be selected. Figure 6A shows the ranking of the experimental genotypes based on the MTSI index, with the genotype having the highest value in the center and the genotype with the lowest value placed in the outermost circle. Applying a selection pressure of 20%, genotype 13 ranked first, and genotypes 10, 8, and 9 were identified as the most ideal stable genotypes across all traits. Comparing the trait values in the selected genotypes based on the MTSI index with the trait values in all experimental genotypes revealed that the mean value of SC increased in the selected genotypes, while the mean values of RY, K^+^, N, and Na^+^ decreased. Increasing SC aligns with the goals of the sugar beet improvement programs, but reducing RY is not one of the objectives. The goals for Na^+^, K^+^, and N are to decrease their values, and the selected genotypes showed a significant reduction in these traits. The selected genotypes resulted in selection differential and favorable selection gain in all traits except RY. Except for RY, the other traits exhibited high heritability in the selected genotypes. Figure 6B provides a visual representation of the strengths and weaknesses of the selected genotypes based on the contribution of each factor in the MTSI index. The lower the share justified by a factor (close to the outer edge), the closer the attributes within that factor are to the stable ideal state. Genotypes 8 and 9, which had the lowest value in the first factor, were close to the ideal genotype for the RY, K^+^, and N. Genotypes 10 and 13 had the lowest share of the second factor, indicating their proximity to the ideal genotype in terms of SC and Na^+^. The MTSI index has been successfully used in previous studies to evaluate yield and other agronomical traits in various genotypes. Sharifi, Abbasian and Mohaddesi^39^ applied the MTSI index to evaluate rice genotypes and identify superior genotypes in terms of yield, yield stability, and other agronomical traits. Similarly, Rajabi et al.^8^ used the MTSI index to identify stable sugar beet genotypes under conditions infected with rhizomania disease. Taleghani et al.^11^ also employed the MTSI index to identify ideal genotypes in terms of RY, WSY, SC, and extraction coefficient of sugar. The findings of these study align with the results obtained, which demonstrate the effectiveness of the MTSI index in identifying superior genotypes.

**Table 8:** Prediction of selection differential for studied traits based on the multi-trait stability index (MTSI).

Variable	FA1	FA2	Communality	Uniqueness’s	Goal	h^2^	SD (%)	SG (%)
Root yield	0.79	0.12	0.64	0.36	Increase	0.57	− 4.03	− 2.28
${{\text{K}}}^{+}$	− 0.83	− 0.10	0.70	0.30	Decrease	0.92	− 3.57	− 3.29
*alpha amino* N	− 0.83	0.39	0.84	0.16	Decrease	0.91	− 3.16	− 2.87
Sugar content	− 0.01	− 0.94	0.89	0.11	Increase	0.91	1.46	1.33
${{\text{Na}}}^{+}$	− 0.02	− 0.92	0.84	0.16	Decrease	0.95	− 14.54	− 13.76
Mean	–	–	0.78	0.22	–	–	–	–
Total decrease	–	–	–	–	–	–	− 25.30	− 22.19
Total increase	–	–	–	–	–	–	1.46	1.33
Eigenvalues	2.09	1.82	–	–	–	–	–	–
Variance (%)	41.76	36.48	–	–	–	–	–	–
Cum. variance (%)	41.76	78.24	–	–	–	–	–	–

FA: Factor, h^2^: Heritability, SD: Selection differential, SG: Selection gain.

## Conclusion

According to the results, the AMMI model demonstrated significant additive and multiplicative effects at the 1% probability level. However, the LMM indicated that the genotype and GEI had significant effects at the same probability level. Consequently, relying solely on the AMMI model for analyzing the structure of the LMM would not be successful. In such situations, utilizing BLUP analysis can yield better and more reliable results due to its high efficiency in estimating the average performance of genotypes in mixed models. By integrating the power of the AMMI method and the prediction accuracy of the BLUP method, it becomes possible to examine genotypic stability and GEI in the form of the WAASB index, while overcoming the limitations of the AMMI model. This comprehensive approach provides a complete picture of the GEI in sugar beet. In the selection and introduction of superior cultivars, it is necessary to consider both stability and yield value. In addition to reducing GEI, genotypes with high yield potential can be selected. Therefore, the WAASBY index can provide useful results. The results demonstrate that the environment and its interaction with the genetic structure of different genotypes significantly influence the phenotypic expression of RY and WSY. This leads to different responses in terms of RY and WSY among genotypes based on varying environmental conditions. In terms of RY values and stability, genotypes 16, 20, 2, and 14 were recognized as stable genotypes with high yield. Similarly, genotypes 20, 3, 16, 4, and 10 exhibited high WSY values and stability. Consequently, these genotypes can be recommended as new genetic sources for sugar beet breeding programs. In such programs, cultivars with high RY, high SC, and low impurities are preferred over other cultivars. The correlation analysis results revealed that all key traits, including SC, Na^+^, K^+^, and N, demonstrated a negative correlation with RY. Genotypes 20, 19, and 16 were identified as the best performers when considering the combination of RY with other key traits. Furthermore, the MTSI identified genotypes 13, 10, 8, and 9 as the most stable genotypes. These genotypes emerged as the top-ranking genotypes, suggesting them as a potential candidate for further breeding programs. In total, this study provides valuable insights into the complex interactions between genotype, environment, and key traits in sugar beet genotypes. This knowledge can guide the development of effective breeding programs aimed at enhancing crop production.

## Data Availability

The data that support the findings of this study are available from the corresponding author upon reasonable request.
